# Geospatial analysis, web-based mapping and determinants of prostate cancer incidence in Georgia counties: evidence from the 2012–2016 SEER data

**DOI:** 10.1186/s12885-021-08254-0

**Published:** 2021-05-06

**Authors:** Justice Moses K. Aheto, Ovie A. Utuama, Getachew A. Dagne

**Affiliations:** 1 Department of Biostatistics, School of Public Health, College of Health Sciences, University of Ghana, P. O. Box LG13, Legon, Accra, Ghana; 2 College of Public Health, University of South Florida, Tampa, USA

**Keywords:** Prostate cancer, Geospatial modelling, Mapping prostate cancer, Disease mapping, R-INLA, SEER program, Georgia, USA

## Abstract

**Background:**

Prostate cancer (CaP) cases are high in the United States. According to the American Cancer Society, there are an estimated number of 174,650 CaP new cases in 2019. The estimated number of deaths from CaP in 2019 is 31,620, making CaP the second leading cause of cancer deaths among American men with lung cancer been the first. Our goal is to estimate and map prostate cancer relative risk, with the ultimate goal of identifying counties at higher risk where interventions and further research can be targeted.

**Methods:**

The 2012–2016 Surveillance, Epidemiology, and End Results (SEER) Program data was used in this study. Analyses were conducted on 159 Georgia counties. The outcome variable is incident prostate cancer. We employed a Bayesian geospatial model to investigate both measured and unmeasured spatial risk factors for prostate cancer. We visualised the risk of prostate cancer by mapping the predicted relative risk and exceedance probabilities. We finally developed interactive web-based maps to guide optimal policy formulation and intervention strategies.

**Results:**

Number of persons above age 65 years and below poverty, higher median family income, number of foreign born and unemployed were risk factors independently associated with prostate cancer risk in the non-spatial model. Except for the number of foreign born, all these risk factors were also significant in the spatial model with the same direction of effects. Substantial geographical variations in prostate cancer incidence were found in the study. The predicted mean relative risk was 1.20 with a range of 0.53 to 2.92. Individuals residing in Towns, Clay, Union, Putnam, Quitman, and Greene counties were at increased risk of prostate cancer incidence while those residing in Chattahoochee were at the lowest risk of prostate cancer incidence.

**Conclusion:**

Our results can be used as an effective tool in the identification of counties that require targeted interventions and further research by program managers and policy makers as part of an overall strategy in reducing the prostate cancer burden in Georgia State and the United States as a whole.

**Supplementary Information:**

The online version contains supplementary material available at 10.1186/s12885-021-08254-0.

## Background

Prostate cancer is the leading diagnosis of malignancy and the second cause of mortality among American men, with an estimated national annual health care cost of $9.8 billion [1, 2]. The United States Cancer Statistics reported 192,443 new cases of prostate cancer in 2016, with an incidence rate of 101 per 100,000 men, and 30,370 prostate cancer deaths or 19 deaths per 100,000 during the same year [3]. Despite an overall decline in incidence across the United States since the early 1990s [4], there remain pockets of high prostate cancer burden.

In the United States, the state of Georgia has the second largest annual incidence rate of prostate cancer [3]. In 2016, there were 7160 reported new cases and 889 deaths in the state, with associated incidence and mortality rates of 133 and 23 per 100,000 men, respectively [3]. African American (AA) men not only have higher incidence of prostate cancer but also demonstrate 60% more mortality than white men, after controlling for incidence [5]. As 32% of Georgia consists of AA [6], it represents an unusual opportunity to investigate community factors associated with a high-risk population. Although a few studies have identified high prostate cancer incidence in the southwest of the state [7, 8], the sociodemographic characteristics of these regions are not well described.

For the purpose of planning for prostate cancer interventions with limited health resources, it is important to characterize and identify predictors of high prostate cancer burden at the community level. The present study, therefore, aims to 1) model and map Georgia county incidence of prostate cancer, 2) evaluate county sociodemographic factors associated with high incidence of prostate cancer.

## Methods

### Data source and study population

We used the Surveillance, Epidemiology, End Results (SEER) population-based cancer registry, which is publicly available data to investigate county-level distribution of prostate cancer cases in the state of Georgia. For this ecological study, only newly diagnosed cases 40 years and older from January 1, 2012 through December 31, 2016 were used for this study, because case reporting to SEER from the greater Georgia area started in 2010 and at the time of analysis SEER’s most current county attributes data spanned the 2012 to 2016 period. The greater Georgia area includes all counties in the state, except the 15 represented by the older Atlanta and Rural Georgia areas previously reported to SEER [9]. Therefore, since 2010 SEER captures cancer data from all 159 counties in Georgia. The SEER Georgia registry reports clinical, or preferentially pathologic diagnosis of cancer from eligible patient records in hospitals, laboratories and physician offices [10, 11]. Patients must be Georgia residents at the time of diagnosis, even though the address of residence is not reported in the registry. Only patients with an International Classification of Diseases for Oncology, third edition, (ICD-O-3) with topography code C61 and behaviour code 3 were included for analysis. SEER, being one of the oldest registries in the country, represents the gold standard in reporting standards and data quality, with completeness rates of more than 97% [12–14].

SEER data are publicly available deidentified records of cancer cases. Permission was sought from and granted by SEER Program to access and use the data for this study. We did not attempt to identify, contact patients or link records to identifiable health information.

### Outcome variable

The outcome variable is the number of incident prostate cancer cases per county. Detailed information is provided under the statistical analysis section.

### Covariates

The covariates used in this study were county-level variables for the period 2012–2016 identified in the literature to be associated with the prostate cancer incidence [2, 15–17]. These included percentage of blacks in the counties, number of persons above 65 years of age in the counties, number of persons having at least a bachelor’s degree in the counties, mean age at diagnosis, number of persons living below poverty in the counties, number of foreign born persons in the counties, percentage of the rural population in the counties, monthly median family income in the counties, and number of unemployed.

### Statistical analysis

We employed a Bayesian geospatial model to investigate both measured and unmeasured spatial risk factors for prostate cancer among men residing in 159 counties in Georgia State.

### Model formulation

We set *Y*_*i*_ to be the observed counts of prostate cancer cases in county *i* and *E*_*i*_ as the expected number of prostate cancer cases in county *i*. We implemented Besag-York-Mollié (BYM) model [18] to analyse the data. We assumed that *Y*_*i*_ are conditionally independently Poisson distributed, and modelled as:

$$ {Y}_i\sim Poisson\left({E}_i{\theta}_i\right),i=1,2,\dots, n $$

where *n* is the number of counties (i.e *n* = 159) and *θ*_*i*_ is the relative risk in county *i*. We expressed the logarithm of *θ*_*i*_ as:

$$ \mathit{\log}\left({\theta}_i\right)={\beta}_0+\boldsymbol{d}{\left({\boldsymbol{x}}_{\boldsymbol{i}}\right)}^{\prime}\beta +{u}_i+{v}_i, $$

where *β*_0_ is the intercept parameter that represents the overall risk, *d*(.) is a vector of observed covariates, *β* is a vector of regression coefficients for the covariates, *u*_*i*_ is a spatial structured effect component. We modelled the *u*_*i*_ using conditional autoregressive (CAR) distribution given as: ${u}_i\mid {\boldsymbol{u}}_{-\boldsymbol{i}}\sim N\left({\overline{u}}_{\delta_i},\frac{\sigma_u^2}{{n\delta}_i}\right),$ and *v*_*i*_ is an unstructured spatial effect defined as ${v}_i=N\left(0,{\sigma}_v^2\right)$.

The relative risk *θ*_*i*_ quantifies whether county *i* has higher (*θ*_*i*_ > 1) or lower (*θ*_*i*_ < 1) risk than the average risk in the reference population. We produced the probabilities of predicted relative risk being greater than a given threshold *c* (exceedance probabilities, i.e. *P*(*θ*_*i*_ > *c*)).

Finally, we visualised the risk of prostate cancer by mapping the predicted relative risk and exceedance probabilities. We developed interactive web-based maps to guide optimal policy formulation and intervention strategies targeted at improving the survival of prostate cancer patients and the overall health of men in Georgia.

Using the Bayesian framework, we implemented our Poisson model through recommended strategies (i.e. Integrated Nested Laplace Approximation (INLA) with Stochastic Partial Differential Equation (SPDE)) [19, 20]. We followed non-informative approach in choosing our priors due to lack of reliable prior information about all parameters, and thus used the default priors available in the R-INLA package. All the analyses were implemented in R-INLA package [21, 22]. We used 95% Bayesian Credible intervals to declare statistical significance.

## Results

### Sample characteristics

On average, 31.6% Georgia county residents were African American or black while the percentage of persons aged ≥65 years was 15.6%. The mean percentage of persons having at least bachelor’s degree in the counties was 17.5% while the overall percentages of persons below poverty and foreign born were 21.6 and 4.6% respectively, and with an average of 60.% rural population among all counties. Overall, the median annual family income was $51,116 and the mean percentage of unemployed was 9.1% (Table 1).

**Table 1:** Georgia county characteristics and crude prostate cancer incident rates, 2012–2016

	% 65+ yrs	% black	% bachelor’s degree	% poverty	% foreign born	% rural population	% unemployed	annual family income	mean annual cases	male population	incidence rate
								median	per county	mean	per 100,000 men
All	15.65	31.69	17.53	21.66	4.61	60.48	9.14	$51,116	40	29,743	158.83
Appling	15.52	0.44	11.92	20.64	4.38	71.44	8.46	$46,350	22	9159	240.20
Atkinson	11.47	42.42	6.68	27.56	13.06	100.00	5.91	$35,000	1	4240	23.58
Bacon	14.35	58.80	13.20	18.30	4.68	69.29	5.29	$46,060	5	5491	91.06
Baker	19.54	21.48	11.00	15.66	5.05	100.00	3.20	$52,280	3	1673	179.32
Baldwin	13.78	51.12	18.42	29.70	2.55	35.14	8.20	$50,230	26	22,683	114.62
Banks	16.24	32.81	10.99	15.52	4.85	93.83	7.66	$50,010	8	9298	86.04
Barrow	11.19	48.78	16.79	14.47	6.91	30.06	8.60	$58,020	42	34,208	122.78
Bartow	12.76	11.24	18.51	14.76	4.68	35.23	7.65	$57,670	59	49,433	119.35
Ben Hill	.	0.21	6.06	.	.	34.00	.	.	12	8439	142.20
Berrien	16.11	55.09	12.27	25.58	3.13	76.14	10.43	$43,070	15	9501	157.88
Bibb	13.94	30.36	24.59	27.79	3.61	14.41	11.32	$50,130	115	73,286	156.92
Bleckley	16.92	59.22	17.72	23.02	1.35	51.59	6.58	$49,520	8	6217	128.68
Brantley	14.42	49.38	9.51	21.18	0.85	99.45	9.15	$43,880	8	9189	87.06
Brooks	18.34	8.11	12.44	24.71	3.45	71.04	17.67	$44,000	12	7901	151.88
Bryan	9.99	5.33	33.34	13.27	4.54	52.34	9.26	$76,470	16	14,852	107.73
Bulloch	10.19	46.74	28.25	31.55	3.47	48.28	9.72	$50,350	28	35,030	79.93
Burke	14.02	29.67	10.40	30.50	2.05	75.00	7.30	$39,800	9	11,186	80.46
Butts	14.45	37.61	10.20	20.53	2.99	77.94	8.35	$53,170	19	12,522	151.73
Calhoun	12.53	32.86	10.29	32.71	4.21	100.00	14.41	$33,330	8	3953	202.38
Camden	11.18	33.55	22.62	13.98	3.74	31.44	9.27	$60,560	35	25,569	136.88
Candler	16.47	55.10	14.23	29.72	4.94	66.97	7.09	$37,140	8	5437	147.14
Carroll	12.41	38.48	18.19	19.26	3.71	41.83	10.80	$55,020	53	53,793	98.53
Catoosa	15.77	24.11	19.65	11.85	2.41	28.10	7.25	$62,270	39	31,028	125.69
Charlton	12.79	60.83	8.99	20.58	8.98	51.02	12.02	$53,970	7	6847	102.23
Chatham	13.59	0.28	32.89	17.99	6.18	4.50	9.25	$61,810	153	127,704	119.81
Chattahoochee	3.85	46.88	30.12	14.30	7.45	29.52	15.96	$47,800	3	7039	42.62
Chattooga	16.16	49.64	8.86	22.40	3.08	57.56	10.06	$41,890	23	13,513	170.21
Cherokee	11.88	44.13	35.50	10.02	8.88	17.10	5.52	$84,420	89	105,874	84.06
Clarke	9.60	25.51	40.75	35.20	10.06	5.86	8.62	$51,160	54	55,388	97.49
Clay	23.89	25.22	7.44	39.81	2.64	100.00	18.94	$35,430	7	1462	478.80
Clayton	8.18	10.80	19.03	24.28	14.19	0.89	12.18	$47,260	130	124,232	104.64
Clinch	15.13	42.17	14.38	35.30	2.88	60.43	11.27	$37,070	10	3315	301.66
Cobb	10.58	31.16	44.99	11.64	15.68	0.25	6.78	$82,200	447	334,369	133.68
Coffee	12.57	25.63	13.08	24.50	5.87	66.58	7.27	$43,440	36	21,455	167.79
Colquitt	13.92	36.89	12.92	24.99	10.60	58.95	7.74	$39,510	22	22,576	97.45
Columbia	11.66	17.14	35.10	9.49	6.97	16.23	6.87	$79,820	66	60,328	109.40
Cook	14.71	33.46	13.84	26.23	2.61	59.41	5.37	$39,560	14	8372	167.22
Coweta	12.47	70.71	28.09	11.98	5.70	32.93	6.57	$74,710	81	62,242	130.14
Crawford	16.62	35.85	13.11	19.08	1.47	100.00	9.64	$48,160	8	6381	125.37
Crisp	15.45	36.08	15.08	32.93	2.65	47.03	13.93	$37,730	15	11,221	133.68
Dade	16.95	42.47	13.80	16.61	2.27	72.13	5.85	$56,020	12	8192	146.48
Dawson	18.19	9.72	29.84	13.42	3.42	80.31	7.47	$69,480	21	11,164	188.10
Decatur	10.33	29.67	41.74	18.99	16.28	56.48	9.76	$62,010	17	13,605	124.95
DeKalb	15.50	22.28	16.89	26.48	2.83	0.26	6.65	$45,580	410	331,355	123.73
Dodge	15.01	37.79	13.54	22.21	1.89	72.23	10.53	$46,660	13	11,449	113.55
Dooly	15.61	34.48	11.26	24.26	3.99	53.67	9.42	$45,240	7	8053	86.92
Dougherty	13.48	35.42	19.77	30.51	2.36	13.96	17.03	$39,890	87	43,927	198.06
Douglas	10.35	29.89	26.15	15.21	8.38	15.76	9.04	$65,010	67	63,772	105.06
Early	18.34	23.10	14.07	31.22	1.50	65.95	7.88	$36,070	10	5191	192.64
Echols	11.13	31.76	7.87	30.21	14.81	100.00	7.04	$50,860	3	2040	147.06
Effingham	10.59	59.60	18.28	10.84	2.71	67.05	5.99	$70,710	22	26,017	84.56
Elbert	18.88	42.31	11.69	19.75	2.24	70.62	8.29	$43,470	17	9656	176.06
Emanuel	15.63	63.49	11.71	29.49	0.98	66.88	11.55	$37,840	25	11,038	226.49
Evans	15.72	27.03	15.10	26.15	4.15	61.28	7.85	$48,520	7	5387	129.94
Fannin	25.49	35.35	17.72	18.00	1.87	100.00	9.23	$50,730	25	11,547	216.51
Fayette	16.04	39.31	45.78	7.14	9.23	18.18	6.35	$96,220	65	51,505	126.20
Floyd	15.50	16.50	19.80	19.75	6.77	36.82	9.28	$53,410	102	46,640	218.70
Forsyth	11.09	42.40	48.27	6.42	14.74	9.92	4.85	$103,920	114	87,194	130.74
Franklin	19.15	5.29	12.64	25.24	3.18	88.93	7.92	$46,800	11	10,911	100.82
Fulton	10.39	17.53	49.81	16.95	12.52	1.08	8.90	$80,420	593	448,267	132.29
Gilmer	21.64	16.52	17.84	19.45	6.91	87.64	7.75	$51,700	25	14,146	176.73
Glascock	16.90	23.58	8.22	15.19	0.66	100.00	6.85	$51,990	1	1487	67.25
Glynn	17.48	45.16	28.16	18.71	5.49	20.57	7.79	$56,320	58	37,855	153.22
Gordon	13.40	36.00	12.92	20.60	9.85	51.56	7.73	$45,890	30	27,283	109.96
Grady	16.28	32.73	12.77	29.62	5.69	62.36	9.18	$40,870	16	12,115	132.07
Greene	25.96	0.74	24.75	24.31	4.95	82.75	6.61	$54,440	22	7809	281.73
Gwinnett	8.59	36.48	34.93	13.02	24.72	0.49	6.85	$69,230	415	397,153	104.49
Habersham	17.70	62.20	17.51	18.33	8.80	58.76	7.22	$50,790	42	20,301	206.89
Hall	13.55	24.21	22.47	17.72	16.54	20.56	5.72	$60,460	134	89,601	149.55
Hancock	19.39	63.74	10.89	31.36	2.66	61.59	10.31	$30,910	11	5170	212.77
Haralson	15.82	32.82	13.76	20.28	1.39	77.36	10.29	$51,340	10	14,072	71.06
Harris	16.13	62.82	26.60	8.38	2.12	96.68	8.08	$81,000	29	15,975	181.53
Hart	20.38	46.10	13.65	21.22	2.60	74.47	5.59	$47,930	24	12,455	192.69
Heard	15.32	12.82	10.50	17.04	0.65	100.00	9.78	$54,820	8	5885	135.94
Henry	10.33	40.86	27.48	12.08	7.44	13.85	8.92	$69,640	103	97,859	105.25
Houston	11.80	58.90	24.03	17.95	5.53	9.96	8.80	$63,930	68	68,066	99.90
Irwin	17.70	20.66	11.15	24.89	0.56	64.71	6.92	$44,210	12	4804	249.79
Jackson	13.42	16.03	19.07	13.52	4.67	60.01	6.77	$62,980	45	30,002	149.99
Jasper	15.29	25.10	10.36	20.06	2.59	81.76	8.77	$45,140	6	6916	86.76
Jeff Davis	.	38.94	8.35	.	.	69.51	.	.	19	7464	254.56
Jefferson	16.88	6.44	10.42	28.91	2.01	80.67	13.28	$41,100	12	8183	146.65
Jenkins	17.74	19.93	13.03	28.32	2.13	66.10	6.22	$41,910	6	3959	151.55
Johnson	15.48	52.27	8.69	25.17	0.68	65.41	9.27	$43,700	7	5592	125.18
Jones	15.77	31.51	20.23	13.69	1.03	67.71	8.05	$64,010	21	13,870	151.41
Lamar	15.95	18.24	17.16	22.17	2.31	60.87	13.04	$51,290	20	8852	225.94
Lanier	12.37	35.79	15.40	28.22	1.56	71.13	13.89	$44,600	9	5084	177.03
Laurens	16.30	36.51	15.24	27.75	2.17	56.64	5.94	$42,940	25	23,066	108.38
Lee	10.64	38.35	24.16	11.91	3.93	36.23	6.93	$72,360	19	14,097	134.78
Liberty	7.49	52.55	18.90	16.93	6.00	23.16	13.00	$46,500	13	30,962	41.99
Lincoln	20.51	0.76	13.15	25.36	1.40	100.00	9.06	$47,840	7	3896	179.67
Long	8.60	1.82	15.24	16.19	6.41	81.34	16.32	$54,780	7	7162	97.74
Lowndes	10.82	47.04	23.86	24.98	4.22	27.20	10.97	$50,800	48	53,285	90.08
Lumpkin	15.72	31.18	26.99	21.64	3.49	83.94	6.79	$51,680	27	14,894	181.28
Macon	14.39	11.11	8.53	32.56	2.59	53.19	16.93	$38,120	9	7973	112.88
Madison	15.88	17.56	15.47	16.10	3.84	91.88	8.01	$53,000	31	13,898	223.05
Marion	17.41	32.91	11.08	25.23	2.38	100.00	14.45	$44,250	12	4305	278.75
McDuffie	15.78	5.49	14.18	26.06	2.74	60.96	8.59	$45,190	18	10,250	175.61
McIntosh	20.12	57.78	13.77	20.13	2.09	74.31	9.93	$54,360	10	6989	143.08
Meriwether	18.40	12.50	10.15	23.71	0.60	83.28	11.18	$46,610	20	10,492	190.62
Miller	19.86	35.23	11.40	25.14	0.10	100.00	7.85	$47,530	11	2929	375.55
Mitchell	14.84	58.54	11.99	29.86	2.63	54.51	16.63	$37,780	27	12,186	221.57
Monroe	16.70	55.71	22.18	13.25	2.19	80.23	9.04	$60,030	13	13,271	97.96
Montgomery	15.54	58.77	15.55	22.82	5.42	98.71	5.77	$47,480	7	4695	149.09
Morgan	18.01	19.52	20.77	13.27	1.79	75.37	6.95	$58,750	18	8636	208.43
Murray	12.97	20.51	10.86	18.83	7.60	70.13	8.89	$46,560	28	19,652	142.48
Muscogee	12.00	12.96	25.00	20.91	5.51	2.98	10.05	$53,730	167	90,870	183.78
Newton	11.78	35.61	19.81	17.04	6.05	31.24	10.57	$57,230	79	47,626	165.88
Oconee	13.49	55.26	46.56	7.14	6.32	50.32	4.22	$85,780	22	16,007	137.44
Oglethorpe	17.30	34.38	16.62	17.91	2.33	99.25	5.61	$52,680	11	7385	148.95
Paulding	9.37	32.32	24.63	10.74	5.17	20.05	6.76	$69,820	73	69,578	104.92
Peach	13.07	25.00	20.15	21.02	5.34	38.22	10.45	$53,280	24	13,416	178.89
Pickens	20.28	59.26	24.76	10.27	2.99	73.10	7.20	$65,680	24	14,440	166.20
Pierce	15.54	31.91	12.90	19.94	2.45	79.35	8.30	$50,720	13	9202	141.27
Pike	14.51	57.78	15.31	12.13	0.91	98.96	10.01	$62,520	14	8742	160.15
Polk	14.88	26.79	12.98	20.14	7.05	51.42	8.73	$48,100	41	20,518	199.82
Pulaski	18.25	37.87	11.81	23.75	1.48	66.70	5.92	$46,830	8	5191	154.11
Putnam	21.31	44.37	18.29	17.76	5.72	80.95	8.03	$56,540	43	10,331	416.22
Quitman	25.49	14.91	8.56	25.68	1.62	73.10	18.53	$34,690	3	1200	250.00
Rabun	25.47	12.50	26.34	21.77	5.75	79.28	6.75	$53,470	18	8025	224.30
Randolph	18.29	56.68	13.35	28.66	2.39	50.63	9.75	$35,570	6	3552	168.92
Richmond	12.56	10.48	21.04	25.19	3.50	9.22	11.48	$46,840	137	97,015	141.22
Rockdale	12.57	5.95	25.96	17.16	9.61	14.93	10.26	$57,620	59	40,533	145.56
Schley	15.31	21.68	14.86	21.87	1.61	100.00	12.96	$47,760	2	2407	83.09
Screven	16.72	40.45	14.38	25.00	1.01	78.92	8.49	$42,460	10	7116	140.53
Seminole	21.30	45.34	14.92	19.08	1.38	68.55	8.74	$43,540	7	4139	169.12
Spalding	16.23	1.69	15.39	23.57	3.45	41.62	10.16	$50,060	63	31,046	202.92
Stephens	18.30	51.67	17.62	20.04	2.10	58.56	10.55	$50,870	19	12,528	151.66
Stewart	15.23	48.43	10.42	41.41	29.13	100.00	13.89	$22,500	3	3682	81.48
Sumter	14.88	22.67	19.95	33.62	3.13	41.78	12.70	$42,090	20	15,627	127.98
Talbot	19.61	20.36	12.65	20.69	0.97	93.88	9.31	$44,730	11	3245	338.98
Taliaferro	22.32	19.69	8.77	31.38	3.41	100.00	11.65	$41,630	2	841	237.81
Tattnall	12.16	0.00	11.37	27.68	3.58	68.24	5.07	$46,550	18	14,860	121.13
Taylor	18.02	61.97	11.32	28.39	1.22	100.00	17.91	$31,880	7	4301	162.75
Telfair	15.20	32.20	9.12	28.70	12.61	46.99	4.28	$30,470	13	9452	137.54
Terrell	17.56	32.26	12.08	34.72	0.59	51.00	12.13	$37,260	15	4479	334.90
Thomas	16.50	16.13	19.53	21.30	2.79	46.02	9.80	$46,330	32	21,179	151.09
Tift	13.67	41.55	17.52	27.48	6.42	40.78	5.09	$45,620	23	19,210	119.73
Toombs	14.99	26.02	17.02	26.56	6.16	51.06	10.83	$44,700	16	12,928	123.76
Towns	33.12	18.20	25.13	15.07	2.76	100.00	8.91	$48,720	19	4996	380.30
Treutlen	17.30	2.07	16.49	18.67	1.24	58.87	6.19	$55,410	3	3449	86.98
Troup	13.58	35.38	18.76	21.32	4.04	44.30	10.74	$52,120	52	32,215	161.42
Turner	18.35	42.67	12.28	27.65	4.31	49.73	9.13	$42,630	6	4358	137.68
Twiggs	19.53	69.23	11.64	30.32	1.08	100.00	7.74	$41,150	12	4398	272.85
Union	31.21	32.04	22.37	13.12	2.15	100.00	9.06	$53,700	26	10,397	250.07
Upson	17.56	38.54	13.35	22.94	1.34	46.91	12.47	$47,500	22	13,024	168.92
Walker	16.57	11.18	15.02	18.44	1.15	43.85	6.99	$51,320	43	33,781	127.29
Walton	14.17	2.97	18.58	13.20	3.86	42.66	7.81	$62,470	66	40,763	161.91
Ware	15.92	55.26	12.83	28.07	3.39	29.44	5.65	$42,150	32	18,069	177.10
Warren	20.48	2.69	12.09	26.38	1.98	100.00	12.54	$39,890	5	2694	185.60
Washington	15.88	46.51	12.30	26.40	1.67	65.60	10.70	$46,630	20	10,812	184.98
Wayne	14.51	32.56	13.32	20.58	2.82	57.94	12.83	$50,680	21	15,719	133.60
Webster	17.51	23.68	9.42	22.41	0.26	100.00	5.20	$51,370	4	1362	293.69
Wheeler	12.96	1.94	4.94	27.41	1.53	100.00	7.53	$36,210	5	4580	109.17
White	19.95	1.06	20.79	19.29	2.75	83.79	4.81	$50,120	18	13,269	135.65
Whitfield	12.71	28.43	13.55	19.63	18.31	29.08	9.63	$49,450	69	51,118	134.98
Wilcox	16.06	1.64	9.53	20.87	2.39	100.00	7.89	$45,350	7	5436	128.77
Wilkes	21.37	9.02	13.79	26.74	3.57	67.37	8.62	$47,480	10	5169	193.46
Wilkinson	17.77	34.09	8.64	20.79	0.77	100.00	7.38	$50,130	8	4582	174.60
Worth	16.90	47.81	10.21	18.44	1.65	69.16	8.23	$45,340	17	10,397	163.51

### Risk factors from non-spatial and spatial models

**Fig. 1:**
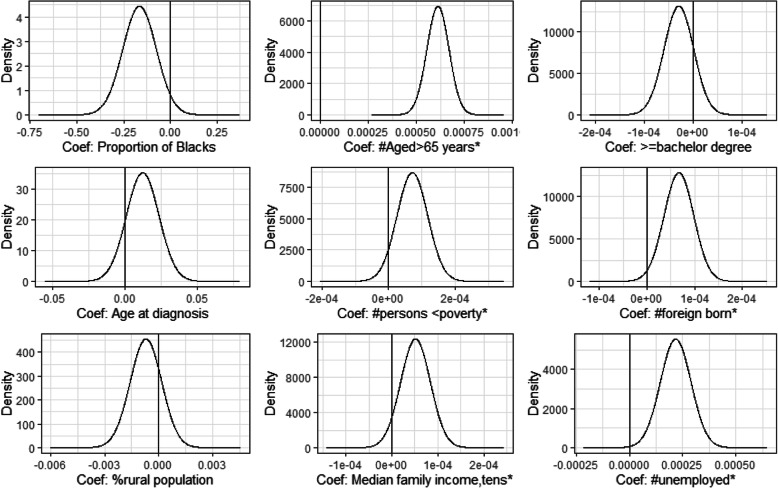
Risk factors associated with prostate cancer incidence in the non-spatial model

Number of persons above age 65 years and below poverty, higher median family income, number of foreign born and unemployed were risk factors independently associated with prostate cancer risk in the non-spatial model (Fig. 1).

**Fig. 2:**
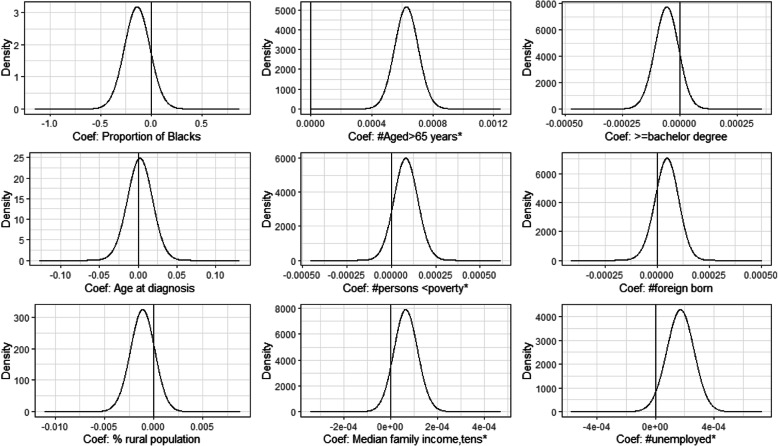
Risk factors associated with prostate cancer incidence in the spatial model

Except for number of foreign born, all these significant risk factors in the non-spatial model were also significant in the spatial model with the same direction of effects (Fig. 2).

### Mapping predicted risk of prostate cancer incidence from the Bayesian spatial model

**Fig. 3:**
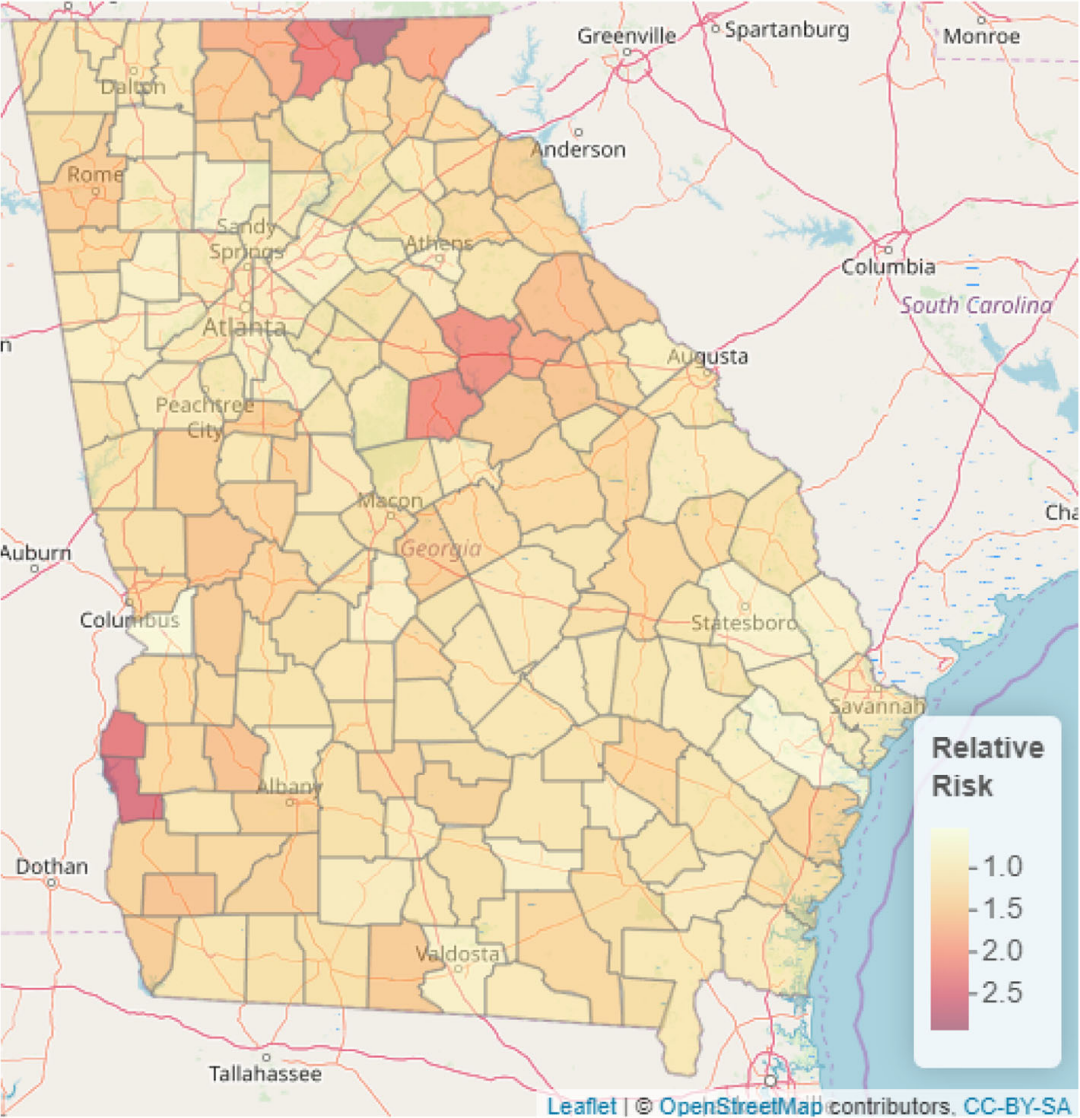
Spatial distribution of predicted prostate cancer relative risk in the Georgia State. Source: This map was produced by the authors

Substantial geographical variations in prostate cancer incidence were found in the study (Fig. 3). In addition, we presented the web-based interactive map of Fig. 3 in the supplementary material online. The predicted mean relative risk (RR) was 1.20 with a range of 0.53 (95% CI: 0.34, 0.78) to 2.92 (95% CI: 2.13, 3.86). Individuals residing in Towns, Clay, Union, Putnam, Quitman, and Greene counties were at increased risk of prostate cancer incidence while those residing in Chattahoochee were at the lowest risk of prostate cancer incidence.

**Fig. 4:**
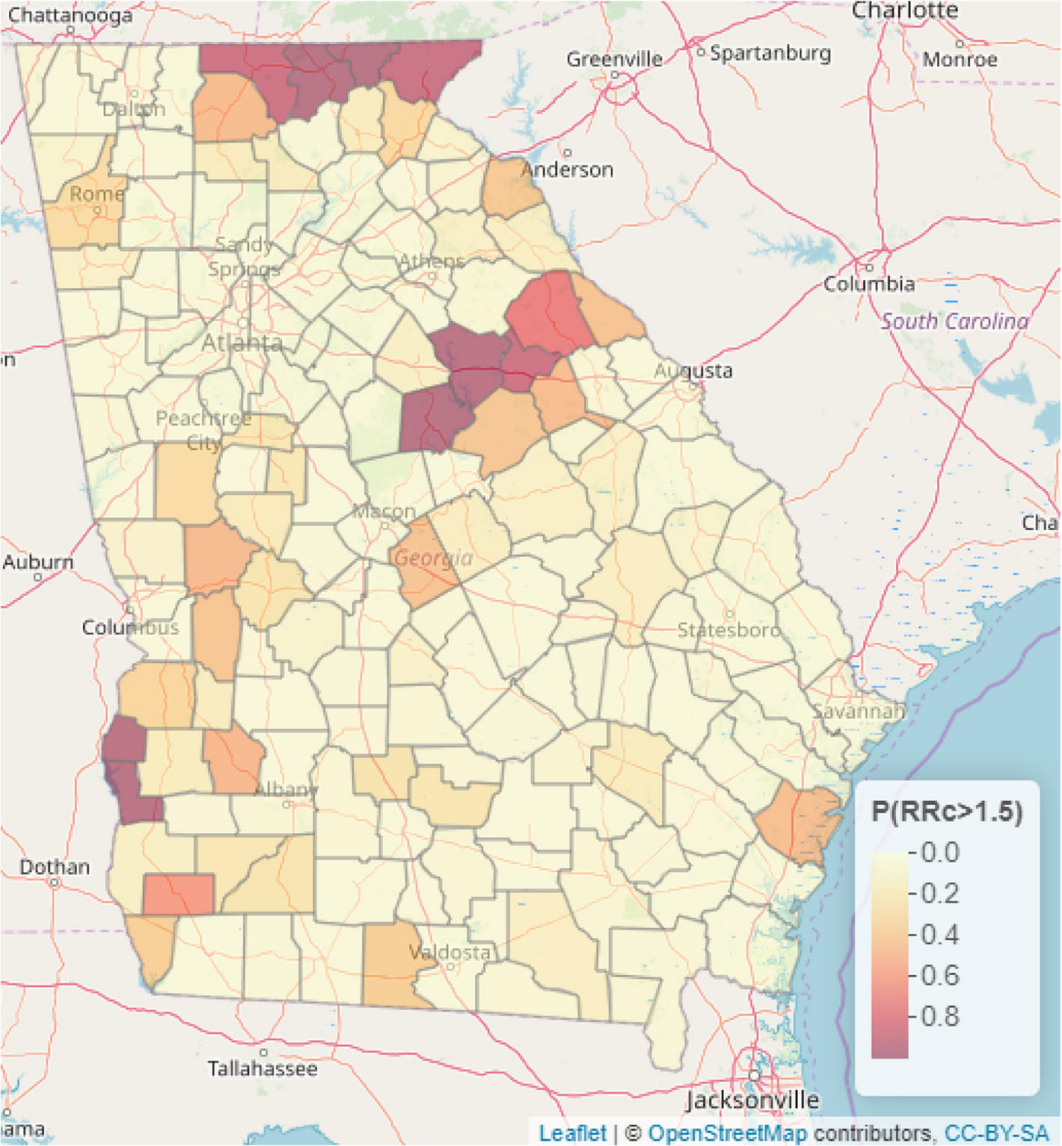
Predictive maps for exceedance probability of relative risk of 1.5 (i.e. P (RR > 1.5)). Source: This map was produced by the authors

**Fig. 5:**
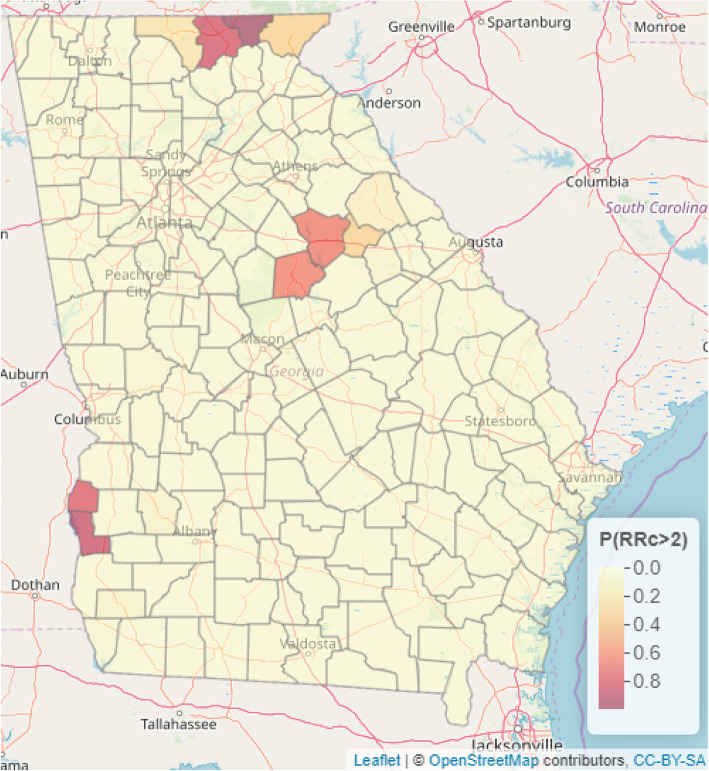
Predictive maps for exceedance probability of relative risk of 2 (i.e. P (RR > 2)). Source: This map was produced by the authors

Presented in Figs. 4 and 5 are the predictive maps of the probability that the relative risk will exceed 1.5 and 2 respectively at a given county in the Georgia State. We also presented the web-based interactive map of Figs. 4 and 5 in the supplementary material online. The deep red regions represent counties where the probability of the relative risk exceeding 1.5 (Fig. 4) and 2 (Fig. 5) are high.

The probability that the relative risk will exceed 1.5 is highest in Union, Towns, Putnam, Greene and Quitman counties (Fig. 4). Also, the probability that the relative risk will exceed 2 is highest in Towns county with a probability of 0.99 (Fig. 5).

## Discussion

The study sets out to use Bayesian geospatial methods to model and map prostate cancer incidence in Georgia counties, and to evaluate county sociodemographic factors associated with high incidence of prostate cancer for the purpose of optimal planning for prostate cancer interventions amidst limited public health resources. Critical risk factors for prostate cancer identified in the present study included number of persons above 65 years of age and below poverty, median family income and number of foreign born and the unemployed in counties. In contrast to previous studies [5, 7], our study did not find an association between prostate cancer incidence and proportions of blacks and rural population.

One of the important aims of this study is identification of high-risk counties for public health interventions amidst limited public health resources. This is critical because residential location of people could act as a marker for the socioeconomic, personal, and climatic/environmental factors that influence access to healthcare services and the general health of the people. Thus, spatial modelling and mapping provides the required tools to obtain an improved understanding of health outcomes of people by place for targeted public health interventions [7, 23–27]. The predicted relative risk ranges from 0.53 (95% CI: 0.34, 0.78) in Chattahoochee to 2.92 (95% CI: 2.13, 3.86) in Towns with a mean of 1.20. The study identified Towns (2.92) as the county with the highest prostate cancer incidence. Other counties with relatively high incidence include Clay (RR = 2.55), Quitman (RR = 2.39), Union (RR = 2.30), Greene (RR = 2.14) and Putnam (RR = 2.13) counties were at increased risk of prostate cancer incidence.

On closer examination of high risk prostate cancer counties, we observed that despite being predominantly white and better educated (25.1% with a Bachelor’s degree) the main driver of risk in Towns County in the north of Georgia was its older population, reporting the largest proportion of persons at least 65 years of age (33.1%). While advancing age is a well-known risk factor for prostate cancer, Clay and Quitman Counties in also suggest that low educational attainment (7.4 and 8.5% with a Bachelor’s degree), high unemployment (18.9 and 18.5%) and individual poverty (39.8 and 25.6%) may be additional risk factors in black communities. Exactly how these socioeconomic indices may impact prostate cancer risk within older black populations is not well known, but high cigarette use and alcohol consumption as well as poor diet have been hypothesized to mediate or moderate this risk [28]. Furthermore, risk factors of exposures to water, air and soil pollution from agricultural farming of cash crops such as cotton, from the southwest through to central Georgia, may also be involved [29]. As neighbouring lower risk counties with large or predominantly black populations likely shared these environmental conditions with Clay and Quitman, our modelling suggests that prostate cancer risk in both communities is multifactorial, resulting from a possible confluence of negative lifestyle, economic and environmental factors experienced over long periods of time.

In comparing the high-risk counties with Chattahoochee and rural low-risk counties, we observed that population age was the single most obvious distinction. Low risk counties had a smaller proportion of elderly persons, irrespective of whether they were classified as rural, and in particular, Chattahoochee had the youngest population (3.8% 65 years and older) with the highest educational attainment (30% with a Bachelor’s degree).

Our study supports the findings of others that reported geographical differences in health outcomes such as prostate and lung cancers, malaria, malnutrition, mortality among others [5, 7, 23–25, 30]. Against the backdrop of a national reduction in incident prostate cancer, there remain pockets of high risk in the north, southwest as well as central areas of Georgia. The present study suggests that there may be racial differences in prostate cancer risk within counties. The aging population may be the main risk factor in overwhelmingly white counties while limited education and poverty may play a larger role in black counties. It should be noted that although several counties with large African American populations were observed to have a high-risk of prostate cancer incidence, the present study found no association between race and prostate cancer risk, in part because these counties tended to be considerably smaller than predominantly white counties. Importantly, this is an ecological study and the associations discussed herein should not be regarded as causal or necessarily significant at the level of individual prostate cancer patients. Prostate Specific Antigen (PSA) screening has driven prostate cancer diagnosis since the 1980s [31, 32]. However, this reliance on PSA has come at the cost of overtreatment and its complications among many low risk men, and in May 2012, the US Prevention Services Task Force (USPSTF) recommended against routine PSA screening for all men [32, 33]. While current diagnostic practices among prostate cancer patients may be of interest and the scope of the present study may represent a substantial post-recommendation period, our study design additionally prevents comparisons that are better made over time among individual patients managed by primary care physicians [32]. Furthermore, we did not include individual-level diagnostic data in our analysis. With these constraints in mind, our results are best suited for hypotheses generation.

### Strengths and limitation

The use of Bayesian spatial analysis methods in this study provided an essential tool for the investigation of prostate cancer incidence in relation to risk factors to help in the better understanding of spatial distribution and potential etiologic mechanism of prostate cancer disease using an internationally recognised gold standard SEER data. Our modelling approach also allowed counties with small counts to borrow information from their neighbouring counties thereby reducing the risk of inflated relative risk due to small expected counts. Furthermore, unlike the frequentist spatial modelling approach, our Bayesian spatial modelling approach allowed graphical presentation of the posterior distribution of risk factor effects on the prostate cancer incidence as presented in Figs. 1 and 2. The present study might have left out some potential risk factors that might explain some of the geographical differences in prostate cancer disease observed in the study so the findings should be interpreted with caution.

Our findings broadly support previous studies [2, 15–17, 34] that report that older ages (≥65 years), income (number below poverty and median family income), race (being a foreign born) and unemployed are critical risk factors for prostate cancer disease. For example, the finding that the number of persons aged 65 years or older increased the risk of the disease supports previous studies that reported that prostate cancer risk increases with age, and with incidence rate over 60% [34–36]. The finding that increased number of foreign born increases the risk of prostate cancer disease supports previous studies that reported prostate cancer inequality by race [7].

## Conclusion

Our modelling approach captured variation in prostate cancer risk over the whole of the Georgia State. The risk maps indicate substantial geographical variations in the risk of prostate cancer. This can be used as an effective tool in the identification of counties that require targeted interventions and further research by program managers and implementers as part of an overall strategy in reducing the prostate cancer burden in the Georgia State and the U.S. as a whole. For example, a further research could aim at identifying as yet unidentified risk factors that might have accounted for the geographical differences we observed in the prostate cancer disease among the counties in the Georgia State after we have accounted for the present risk factors in our model.

Furthermore, we advocate for implementation of focused strategies to decrease prostate cancer incidence and to improve survival in the presence of the identified critical risk factors in this study.

## Supplementary Information

**Additional file 1.**

**Additional file 2.**

**Additional file 3.**

## Data Availability

Data is freely available upon making official request to Surveillance, Epidemiology, and End Results (SEER) Program through the website at https://seer.cancer.gov/**.**

## Abbreviations

AA: African American
CI: Credible Interval
ICD-O-3: International Classification of Diseases for Oncology, third edition
INLA: Integrated Nested Laplace Approximation
RR: Relative Risk
SEER: Surveillance, Epidemiology, and End Results
SPDE: Stochastic Partial Differential Equation
U.S.: United States of America

## References

[1] Roehrborn CG, Black LK (2011). The economic burden of prostate cancer. BJU Int.

[2] Siegel RL, Miller KD, Jemal A (2019). Cancer statistics, 2019. CA Cancer J Clin.

[3] CDC:Centers for Disease Control and Prevention (2019). United States Cancer Statistics.

[4] Kelly SP, Rosenberg PS, Anderson WF, Andreotti G, Younes N, Cleary SD, Cook MB (2017). Trends in the incidence of fatal prostate Cancer in the United States by race. Eur Urol.

[5] Wagner SE, Hurley DM, Hébert JR, McNamara C, Bayakly AR, Vena JE (2012). Cancer mortality-to-incidence ratios in Georgia: describing racial cancer disparities and potential geographic determinants. Cancer.

[6] USCB: United States Census Bureau (2020). State and County Quick Facts: Georgia.

[7] Wagner SE, Bauer SE, Bayakly AR, Vena JE (2013). Prostate cancer incidence and tumor severity in Georgia: descriptive epidemiology, racial disparity, and geographic trends. Cancer Causes Control.

[8] McNamara C, Davis V, Bayakly AR, Moon T (2010). Prostate Cancer in Georgia, 2002-2006.

[9] SEER: Surveillance Epidemiology and End Results Program (2020). Georgia Center for Cancer Statistics.

[10] Ruhl J, Adamo M, Dickie L (2016). SEER Program Coding and Staging Manual 2016: Section V.

[11] Scosyrev E, Messing J, Noyes K, Veazie P, Messing E (2012). Surveillance epidemiology and end results (SEER) program and population-based research in urologic oncology: an overview. Urol Oncol.

[12] Duggan MA, Anderson WF, Altekruse S, Penberthy L, Sherman ME (2016). The surveillance, epidemiology, and end results (SEER) program and pathology: toward strengthening the critical relationship. Am J Surg Pathol.

[13] Park HS, Lloyd S, Decker RH, Wilson LD, Yu JB (2012). Overview of the surveillance, epidemiology, and end results database: evolution, data variables, and quality assurance. Curr Probl Cancer.

[14] Zippin C, Lum D, Hankey BF (1995). Completeness of hospital cancer case reporting from the SEER program of the National Cancer Institute. Cancer.

[15] ACS: American Cancer Society (2019). Cancer Statistics Center: Georgia.

[16] Lund Nilsen TI, Johnsen R, Vatten LJ (2000). Socio-economic and lifestyle factors associated with the risk of prostate cancer. Br J Cancer.

[17] Hastert TA, Beresford SA, Sheppard L, White E (2015). Disparities in cancer incidence and mortality by area-level socioeconomic status: a multilevel analysis. J Epidemiol Community Health.

[18] Besag J, York J, Mollié A (1991). Bayesian image restoration with applications in spatial statistics (with discussion). Ann Inst Stat Math.

[19] Lindgren F, Rue H, Lindström J (2011). An explicit link between Gaussian fields and Gaussian Markov random fields: the stochastic partial differential equation approach. J R Stat Soc B Stat Meth.

[20] Rue H, Martino S, Chopin N. Approximate Bayesian inference for latent Gaussian models by using integrated nested Laplace approximations. J R Stat Soc B Stat Meth. 2009;71:319–92.

[21] Lindgren F, Rue H (2015). Bayesian Spatial Modelling with R-INLA. J Stat Softw.

[22] Rue H, Martino S, Lindgren F, Simpson D, Riebler A, Krainski E (2014). INLA: Functions Which Allow to Perform a Full Bayesian Analysis of Structured Additive Models Using Integrated Nested Laplace Approximaxion. R package version 00-1404466487.

[23] Aheto JMK, Taylor BM, Keegan TJ, Diggle PJ (2017). Modelling and forecasting spatio-temporal variation in the risk of chronic malnutrition among under-five children in Ghana. Spat Spatiotemporal Epidemiol.

[24] Aheto JMK (2019). Predictive model and determinants of under-five child mortality: evidence from the 2014 Ghana demographic and health survey. BMC Public Health.

[25] Diggle P, Moyeed R, Rowlingson B, Thomson M (2002). Childhood malaria in the Gambia: A case-study in model-based geostatistics. J R Stat Soc C Appl Stat.

[26] Kandala NB, Madungu TP, Emina JB, Nzita KP, Cappuccio FP (2011). Malnutrition among children under the age of five in the Democratic Republic of Congo (DRC): does geographic location matter?. BMC Public Health.

[27] Nykiforuk CI, Flaman LM (2011). Geographic information systems (GIS) for health promotion and public health: a review. Health Promot Pract.

[28] Brotherton L, Welton M, Robb SW (2016). Racial disparities of pancreatic cancer in Georgia: a county-wide comparison of incidence and mortality across the state, 2000-2011. Cancer Med.

[29] Blomme C, Roubal A, Givens M, Johnson S, Brown L (2020). Georgia County Health Rankings State Report 2020.

[30] Mokdad AH, Dwyer-Lindgren L, Fitzmaurice C, Stubbs RW, Bertozzi-Villa A, Morozoff C, Charara R, Allen C, Naghavi M, Murray CJL (2017). Trends and patterns of disparities in Cancer mortality among US counties, 1980-2014. JAMA.

[31] Catalona WJ, Smith DS, Ratliff TL, Dodds KM, Coplen DE, Yuan JJ, Petros JA, Andriole GL (1991). Measurement of prostate-specific antigen in serum as a screening test for prostate cancer. N Engl J Med.

[32] Cohn JA, Wang CE, Lakeman JC, Silverstein JC, Brendler CB, Novakovic KR, McGuire MS, Helfand BT (2014). Primary care physician PSA screening practices before and after the final U.S. Preventive Services Task Force recommendation. Urol Oncol.

[33] Jemal A, Fedewa SA, Ma J, Siegel R, Lin CC, Brawley O, Ward EM (2015). Prostate Cancer incidence and PSA testing patterns in relation to USPSTF screening recommendations. JAMA.

[34] Gann PH (2002). Risk factors for prostate cancer. Rev Urol.

[35] Rawla P (2019). Epidemiology of prostate Cancer. World J Oncol.

[36] Merriel SWD, Funston G, Hamilton W (2018). Prostate Cancer in primary care. Adv Ther.

